# Higher Content but Not Activity of Stromelysin-2 (MMP-10) in Comparison to Stromelysin-1 (MMP-3) in Human Renal Carcinoma

**DOI:** 10.3390/ijerph191912613

**Published:** 2022-10-02

**Authors:** Jacek Kudelski, Grzegorz Młynarczyk, Monika Gudowska-Sawczuk, Barbara Mroczko, Barbara Darewicz, Marta Bruczko-Goralewska, Lech Romanowicz

**Affiliations:** 1 Department of Urology, Medical University of Bialystok, M. Skłodowskiej-Curie 24A St., 15-276 Bialystok, Poland; 2 Department of Medical Biochemistry, Medical University of Bialystok, Adama Mickiewicza 2C St., 15-089 Bialystok, Poland; 3 Department of Biochemical Diagnostics, Medical University of Bialystok, Waszyngtona 15A St., 15-269 Bialystok, Poland; 4 Department of Neurodegeneration Diagnostics, Medical University of Bialystok, Waszyngtona 15A St., 15-269 Bialystok, Poland

**Keywords:** renal carcinoma, stromelysin, matrix metalloproteinase, MMP-3, MMP-10

## Abstract

Stromelysin-1 and stromelysin-2 (matrix metalloproteinase 3; MMP-3 and matrix metalloproteinase 10; MMP-10, respectively) are enzymes that activate other metalloproteinases. Apart from collagen, they also degrade elastin, fibronectin, gelatin and laminin. In carcinogenic processes, they are involved in angiogenesis and metastasis. Therefore, the aim of this study was to evaluate the DNA content, expression and activity of both stromelysines in cancers of human kidney. Renal carcinoma tissue samples were analyzed. Low- and high-grade cancer tissues were collected. Control material was collected from part of the kidney opposite to the tumor. DNA content, stromelysines content and stromelysin-1 and stromelysin-2 activity were measured using ELISA and Western blot methods. A higher content of deoxyribonucleic acid in low- and high-grade cancer tissues in comparison to the respective control tissue was observed. Both stromelysines were presented in control and cancer tissues in high-molecular-weight complexes. The content of MMP-10 was significantly higher in comparison to MMP-3 in all investigated tissues. Moreover, the content of stromelysin-2 was significantly higher in high-grade (G3) tissues compared to grade 2 (G2) kidney cancer. A significant decrease in the actual and specific activities of both stromelysines was observed with the increase in renal cancer grade. The presented results may indicate that the degradation of extracellular matrix increases with a higher grade of cancer. Moreover, the elevated content and decreased specific activity of stromelysin-2 in cancer tissue indicate that MMP-10 is mainly present in an inactive form in renal carcinoma. Detailed knowledge of the mechanism and participation of stromelysines in extracellular matrix degradation may be important in understanding the pathomechanism of renal cancer development. Therefore, the potential application of stromelysines in the monitoring or prognosis of kidney cancer should be discussed.

## 1. Introduction

The kidneys are parenchymal and paired organs located in the abdominal retroperitoneal space. The most frequent kidney tumor is clear-cell renal cell cancer (ccRCC). The gold standard of kidney cancer treatment is still surgery, but in some advanced cases, patients may also qualify for adjuvant therapy, including the use of nivolumab. However, it is indisputable that both types of treatment may affect the quality of life of patients, which should be always taken into account by clinicians and researchers [1,2]. 

Proper kidney function depends, among other factors, on the adequate structure of the extracellular matrix (ECM). The ECM is a very dynamic and highly charged structure that supports the cells and is an active component in cell signaling [3]. It consists of collagens (main extracellular protein), glycoproteins and elastin molecules that form a complex network, interacting with the surrounding cells. Most of the enzymes engaged in the process of ECM homeostasis maintenance are matrix metalloproteinases (MMPs). MMPs participate in several processes of ECM remodeling as well as in processes of basement membrane destruction, angiogenesis, cell migration and cell apoptosis [4,5,6,7,8]. One group of MMPs are stromelysines: MMP-3 (stromelysin-1), MMP-10 (stromelysin-2) and MMP-11 (stromelysin-3). Stromelisyn-1 shows the highest proteolytic activity and the greatest ability to activate other inactive metalloproteinases such as zymogens [9,10,11]. Despite various forms of collagen, stromelysin-1 also degrades aggrecan, elastin, fibronectin, gelatin, laminin, proteoglycans, and MMP-7, -8 and -13. Importantly, stromelysin-1 is secreted from cells as an inactive form. MMP-3 also has a higher proteolytic potential than MMP-10. Stromelysin-2 degrades collagen types III, IV and V, as well as aggrecan, elastin, fibronectin, gelatin, laminin, MMP-1 and -8 [12,13,14].

It was shown that an increase in MMP-3 expression in tumor cells positively correlated with an increased expression of MMP-1 [15]. Moreover, it was observed that MMP-10 is present in the cytoplasm of renal cell carcinoma cells. This is associated with the local aggressiveness and the degree of tumor malignancy, whereas its expression did not correlate with the tumor size. Stromelysines are considered to be a potential therapeutic target to inhibit the invasion and metastases of renal cell carcinoma [16]. However, the role of stromelisynes in renal carcinoma is not yet fully explained. Therefore, the aim of this study was to evaluate the DNA content, expression and activity of stromelysin-1 and stromelysin-2 in cancers of human kidney.

## 2. Materials and Methods

The study protocol was approved by the Bioethical Committee of the Medical University of Bialystok.

### 2.1. Tissue Material

Tissues were taken immediately after surgery and underwent histopathological examination. The sample population consisted of patients diagnosed with G2 (n = 10) or G3 (n = 10) clear-cell kidney cancer according to the ISUP/WHO malignancy scale. In the investigated material, due to carcinogenetic processes, only structural damage was diagnosed. None of the patients was diagnosed with acute or chronic renal disease. All patients underwent an open nephrectomy. Six female and fourteen male patients were selected for this study, with an age range of 48–78 years and average age of 61 years. For comparison, tissue samples of the same kidney, but always from the side opposite to the tumor, were collected. The control tissues were histologically unchanged. All samples were washed in a 0.9% NaCl solution, weighed, portioned and stored at −70 °C throughout the analysis. Any surgical procedures were performed at the Department of Urology, University Hospital in Bialystok.

### 2.2. Specimen Preparation

Tissue samples were washed with 0.9% NaCl, cut into small pieces and used to prepare a homogenate. Tissue homogenates (20%, *w*/*v*) were prepared in 0.05 M Tris/HCl buffer, pH 7.4, using a knife homogenizer (20,000 rpm, 3 × 30 s, 0 °C). The homogenates were subject to ultrasonification (20 kHz, 3 × 15 s, 0 °C) and centrifugation at 10,000× *g* for 30 min at 4 °C. The supernatants (tissue specimens) were taken, divided and stored at −70 °C for further use.

### 2.3. DNA Content

The DNA content in investigated tissues was measured with the method described by Burton [17].

### 2.4. Contents of Stromelysines 

The contents of stromelysin-1 and stromelysin-2 were evaluated using ELISA method (MMP-3 ELISA Kit for Cell and Tissue Lysates provided by Sigma; cat. no RAB0362 and MMP-10 ELISA Kit provided by Cloud-Clone Corp.; cat. no SEA099Hu, respectively) according to manufacturers’ instructions.

### 2.5. Western Blot of Stromelysines

The 20 μg samples of protein of stromelysin-1 and 20 μg of protein for stromelysin-2 of examined tissue specimens were electrophoresed on 10% SDS-polyacrylamide gel using the method of Laemmli [18] in non-reducing and reducing conditions and blotted to nitrocellulose membranes (Sigma-Aldrich; Saint Louis, MO, USA) at 100 mA for 1 h. Then, 5% (*w*/*v*) nonfat powdered milk in TBS-T solution (20 mM Tris/HCl buffer, pH 7.4, 150 mM NaCl, 0.05% (*v*/*v*) Tween 20) was used for 1 h to block the membranes. Subsequently, they were incubated overnight at 4 °C with a specific antibody directed against human stromelisin-1 (cat. no. MAB905; R&D Systems, Minneapolis, MN, USA) and specific antibody against stromelysin-2 (cat. no. MAB910; R&D Systems, USA) in TBS-T, containing 1% bovine serum albumin (*w*/*v*). Following several washes, bounded antibodies were detected using alkaline phosphatase-conjugated with respective secondary antibody in the same solution for 1 h at room temperature with gentle mixing and the next BCIP/NBT reagent (catalogue number B1911; Sigma). The estimation of molecular mass of stromelysines was carried out by means of pre-stained molecular mass markers (BioRad, Berkeley, CA, USA).

### 2.6. Activity of Stromelysines

The measurements of the actual and specific activities of stromelysin-1 and stromelysin-2 were was conducted in a black flat-bottom 96-well microplate, which was pre-coated with the respective specific antibody (the same antibodies were used in the Western blot analysis; Shi et al., 2009) [19]. Next, 10 µL of the sample was added to each well to immobilize the stromelysin. Then, the microplate was incubated overnight at 4 °C. All other proteins were washed out using the TBS-T buffer (50 mM Tris/HCl pH 7.4, 0.9% NaCl, 0.05% Tween 20). The activity of the stromelysines was measured in 100 μL of 50 mM Tris/HCl buffer pH 7.5 containing 10 mM CaCl_2_, 150 mM NaCl and 0.025% Brij 35 (Watkins et al., 2009), with MCA-Pro-Leu-Ala-Cys(p-OMeBz)-Trp-Ala-Arg(Dpa)-H2 (Merck, cat. No 444258) as a fluorogenic substrate (4 μM final concentration). Furthermore, the microplate was incubated for 1 h at 37 °C. Then, 25 μL of 100 mM EDTANa_2_ stopped the reaction. A spectrophoto-fluorimetric/multimode microplate reader (Tecan Infinite^®^ 200 PRO; Tecan, Männedorf, Switzerland) was used for the measurement of the degradation of the substrate. The calculation of the quantity of the degraded substrate was based on the calibration curve prepared in the same condition with 7-amino-4-methylcoumarin (cat. no 257370; Sigma-Aldrich, Saint Louis, MO, USA) as a standard. The specific activity of stromelysine was expressed in katals/kg of protein.

### 2.7. Protein Determination

The measurement of the protein concentration was carried out according to the Bradford method [20].

### 2.8. Statistical Analysis

The results are presented as mean values ± standard deviations (SD). The statistical analysis of the results was performed using Student’s *t* test. *p* values less than 0.05 were considered significant. We performed statistical analysis using Statistica 10 (StatSoft Polska, Cracow, Poland)

## 3. Results

### 3.1. DNA Content

**Figure 1:**
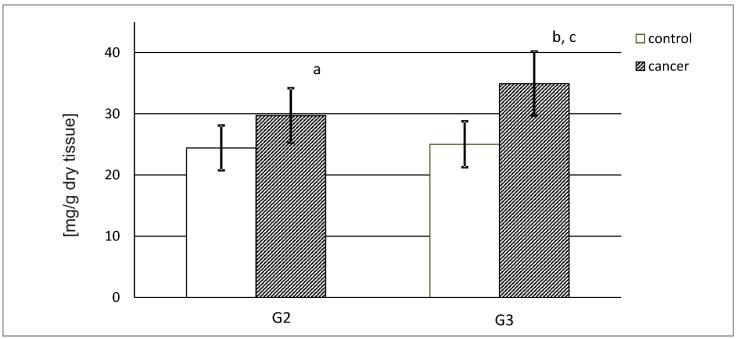
DNA content in control tissue and G2 and G3 (HG) kidney cancer. a—*p* < 0.05 G2 vs. kidney control b—*p* < 0.001 G3 vs. kidney tumor c—*p* < 0.05 G3 vs. G2.

Figure 1 shows the DNA content in the control kidney and for both grades of renal cancer in milligrams, recalculated per gram of dry tissue. The control urinary bladder contains approximately 25 mg of DNA. G2 and G3 of cancer present a higher content of DNA. In the G2 cancer, an increase was observed in more than 21% of DNA content compared to the control tissue. The G3 cancer tissue presented an almost 40% increase in DNA content in comparison with the control tissue (Figure 1).

### 3.2. Stromelysin-1 and Stromelysin-2 Contents

There were differences in the contents of both stromelysines for control human kidney and renal cancer tissue.

**Figure 2:**
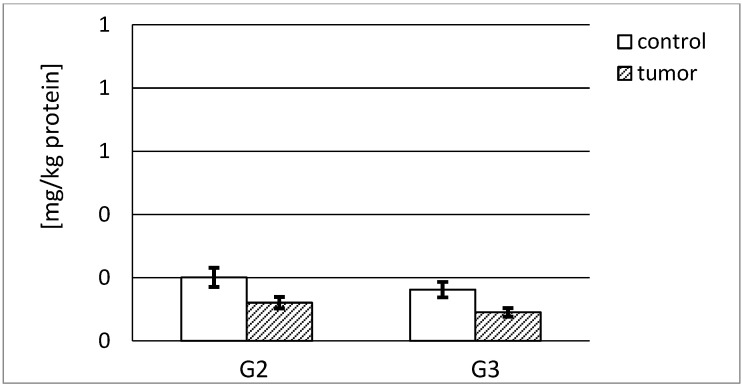
Content of MMP-3 in respective control tissue and G2 grade and G3 grade human kidney tumor. *p* < 0.001—cancer vs. respective control kidney; *p* < 0.001—G3 grade vs. G2 grade human kidney cancer.

Stromelysin-1 was present in a low amount in normal tissue samples (0.201 and 0.162 mg/kg protein). However, the G2 and G3 tumor samples contained considerably lower amounts of stromelysin-1. Almost 40% less of the enzyme was found in the G2 tumor. Furthermore, the G3 tumor contained about 45% less of the enzyme compared to the respective control tissue. In addition, the tumor grade progression from G2 to G3 entailed a significant decrease in stromelysin-1 content (Figure 2).

**Figure 3:**
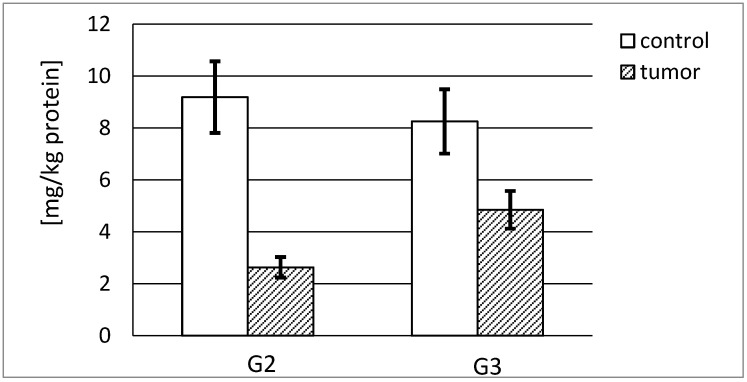
Content of MMP-10 in control tissue and G2 grade and G3 grade human kidney tumor. *p* < 0.001—cancer vs. respective control kidney; *p* < 0.001—G3 grade vs. G2 grade human kidney cancer.

Normal kidneys contained considerably higher amounts of stromelysin-2 (Figure 3) in relation to stromelysin-1, namely about 45–50 times higher per kg of total protein content. The G2 and G3 renal tumor stages were distinguished by a substantial increase in stromelysin-2 content, but these amounts were still significantly lower compared to their respective control tissues. In G2 tumor samples, the decrease in MMP-10 content was equal to 71%, and in G3 tumor samples it was about 41% in comparison with the respective control tissue. Similar to MMP-3, the reduction in stromelysine 2 content considerably increased with the growing tumor invasiveness (Figure 3).

### 3.3. Expression

#### 3.3.1. Expression of MMP-3

**Figure 4:**
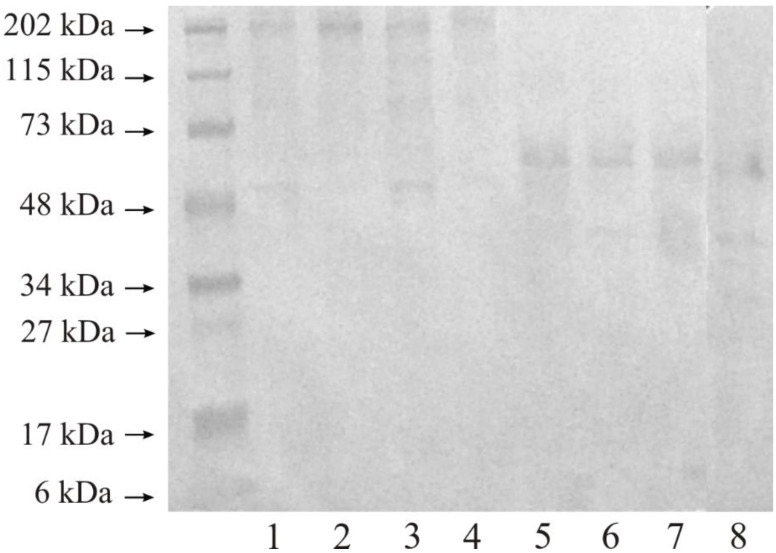
Western immunoblot of MMP-3 in respective control tissue and G2 grade and G3 grade human kidney tumor. Twenty micrograms of protein were applied to the gel. Separation in non-reducing conditions: Lane 1—control kidney for G2 grade tumor, 2—G2 grade of kidney tumor, 3—control kidney for G3 grade tumor, 4—G3 grade of kidney tumor. Separation in reducing conditions: Lane 5—control kidney for G2 grade tumor, 6—G2 grade of kidney tumor, 7—control kidney for G3 grade tumor, 8—G3 grade of kidney tumor.

Figure 4 presents the representative blot for the presence of stromelysine 1 in the control sample compared to the tumor kidney tissue. All studied tissues showed that MMP-3 in high-molecular-weight complexes stayed at the top of the gel—an intensive band whose molecular weight was ca. 202 kDa in non-reducing conditions; lane 1–4. Moreover, both control tissues and both renal cancer grades contained bands with a molecular weight of approx. 73 kDa and 48 kDa. We did not find any other bands with lower masses in non-reducing conditions (Figure 4).

As a result of disulphide bridge reduction with β-mercaptoethanol, high-molecular-weight complexes reacting with the anti-human MMP-3 antibody disappeared in all investigated tissue samples; Figure 4, lane 5–8. Both control and tumor samples contained a band with a molecular weight of approximately 55 kDa. A weak band with a molecular weight of 40 kDa, lane 5–8, was present in all four tissue samples (Figure 4).

#### 3.3.2. Expression of MMP-10

**Figure 5:**
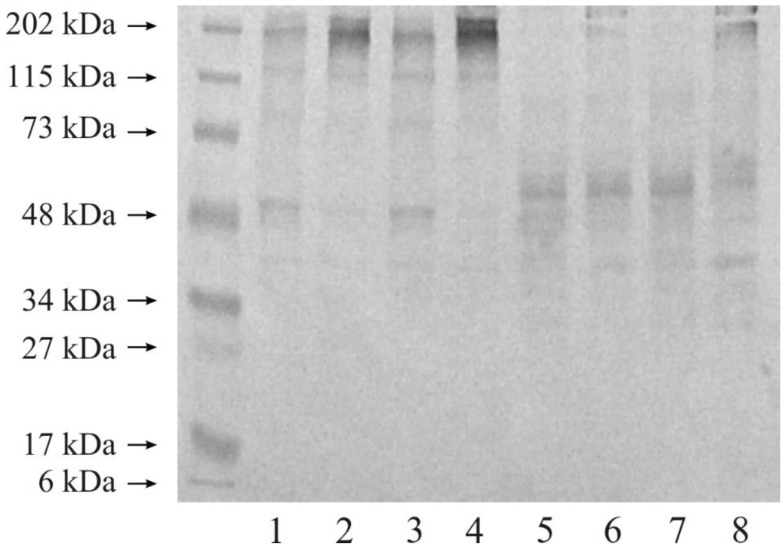
Western immunoblot of MMP-10 in respective control tissue and G2 grade and G3 grade human kidney tumor. Twenty micrograms of protein were applied on the gel. Separation in non-reducing conditions: Lane 1—control kidney for G2 grade tumor, 2—G2 grade of kidney tumor, 3—control kidney for G3 grade tumor, 4—G3 grade of kidney tumor. Separation in reducing conditions: Lane 5—control kidney for G2 grade tumor, 6—G2 grade of kidney tumor, 7—control kidney for G3 grade tumor, 8—G3 grade of kidney tumor.

According to Figure 5, even a low protein load on the gel such as 20 micrograms revealed wide bands at 202 kDa, 115 kDa; weak bands at 73 kDa; and quite wide bands at 48 kDa in all investigated samples (lane 1–4), without a reduction in disulphide bonds. Dark lanes show that the band of 115 kDa indicated the occurrence of MMP-13 in higher mass complexes in both normal tissues and tumor samples. Once the disulphide bonds were reduced, just two bands remained in the Western blot (lane 5–8) and were both clearly separated and present in all these tissues. The molecular weights were ca. 55 kDa and 40 kDa (Figure 5).

### 3.4. Activity of Stromelysin-1 and Stromelysin-2

We measured the actual activity of both stromelysines with the aid of the fluorimetric method with the oligopeptide used as a substrate. In the process, each enzyme was isolated on a microplate pre-coated with an antibody specific to the assayed enzyme. After the immobilization of stromelysines, all other proteins from the tissue sample were washed out. Subsequently, the fluorogenic substrate was added, and the degradation product amount was measured.

#### 3.4.1. Actual and Specific Activity of Stromelysin-1

**Figure 6:**
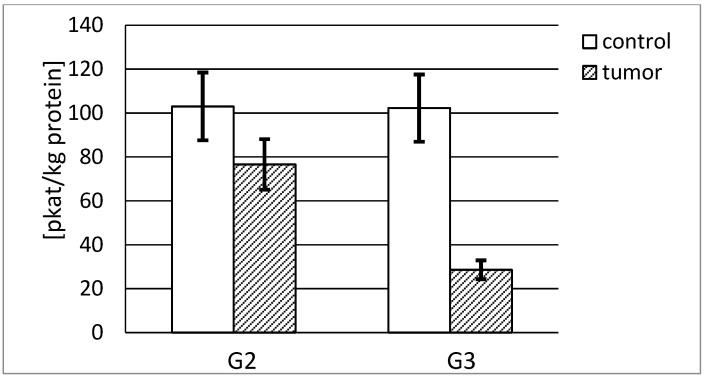
MMP-3 actual activity in human kidney tumor (G2 grade and G3 grade) and respective control tissue. *p* < 0.001—cancer vs. respective control kidney; *p* < 0.001—G3 grade vs. G2 grade human kidney cancer.

Figure 6 shows that a high actual activity of stromelysin-1 was observed in normal human kidney irrespective of its source. The G2 tumor tissue showed a significantly lower actual activity in relation to the respective control tissue, calculated in pikokatals per kg of the total protein content. The G3 tumor presented the lowest stromelysin-1 actual activity, approximately 28% of the activity of respective control tissue. The activity decreased with the increasing grade of renal cancer. The difference between stromelysin-1 activities in both cancer tissue grades was significant. In the case of the G2 tumor, the actual activity of stromelysin-1 was more than 2.5 times higher in comparison with the G3 tumor (Figure 6).

**Figure 7:**
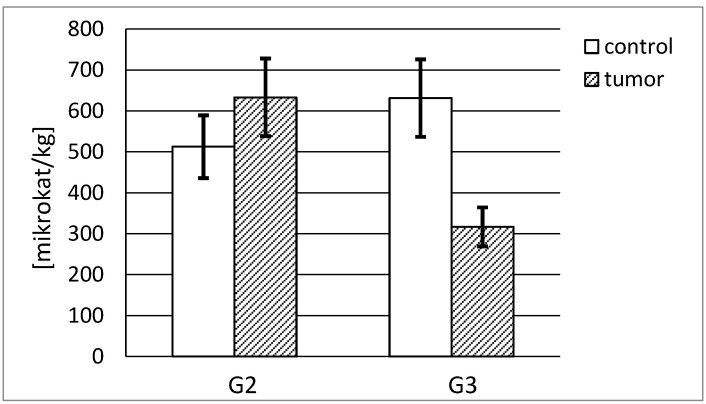
MMP-3 specific activity in human kidney tumor (G2 grade and G3 grade) and respective control tissue. *p* < 0.001—cancer vs. respective control kidney; *p* < 0.001 G3 grade vs. G2 grade human kidney cancer.

The actual specific activity of stromelysin-1 was calculated in mikrokatals per kg of the protein. Both control tissues demonstrated a similar specific activity, 512 and 631 microkatals per kg of stromelysine-1, respectively. The highest activity, almost 20% higher than in the control sample, was found in the G2 tumor tissue. The increase in the grade of renal cancer was accompanied by a decrease in measured activity. There was a significant difference between MMP-3 activities in both grades of cancer tissue. The G2 tumor demonstrated a 2-fold higher specific activity of stromelysin-1 compared with the G3 tumor (Figure 7).

#### 3.4.2. Actual and Specific Activity of Stromelysin-2

**Figure 8:**
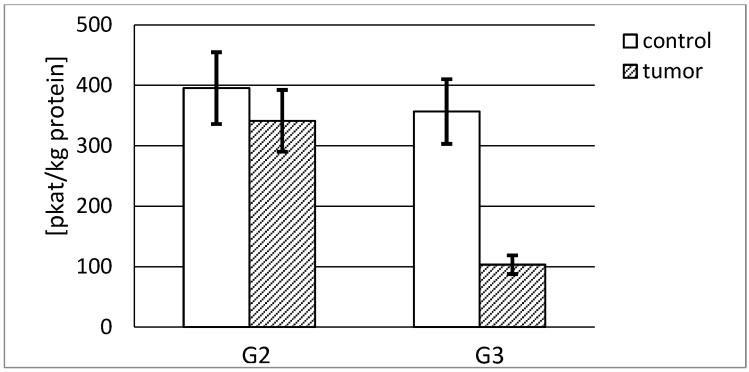
MMP-10 actual activity in human kidney tumor (G2 grade and G3 grade) and respective control tissue. *p* < 0.001—cancer vs. respective control kidney; *p* < 0.001—G3 grade vs. G2 grade human kidney cancer.

Figure 8 demonstrates that the highest actual activity of stromelysin-2 was found in the normal human kidney. The measured enzyme activity decreased with the increase in kidney cancer grade. Significant differences in actual activity of stromelysin-2 expressed in pikokatals per kg of total protein between both tumor grades were found. It was nearly 3.5 times higher for the G2 tumor than for the G3 stage (Figure 8). 

**Figure 9:**
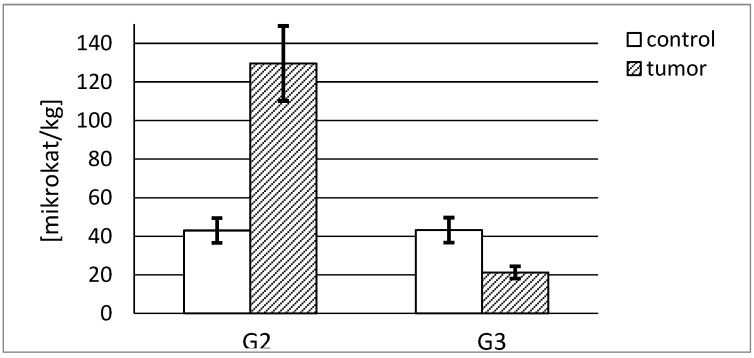
MMP-10 specific activity in human kidney tumor (G2 grade and G3 grade) and respective control tissue. *p* < 0.001—cancer vs. respective control kidney; *p* < 0.001 G3 grade vs. G2 grade human kidney cancer.

As a result of the conducted measurement of stromelysin-2 content in evaluated tissues, we were able to calculate the actual specific activity of stromelysin-2 in mikrokatals per kg of the enzyme protein. As can be seen in Figure 9, that activity was three times higher in the G2 tumor tissue compared to the respective control tissue (Figure 9).

## 4. Discussion

The kidney is a parenchymal organ characterized by a low amount of extracellular matrix. The ECM contains not only collagen, but also many other, equally important components, such as elastin, several proteoglycans and structural glycoproteins. A special layer of ECM separating the epithelial tissue from the connective tissue is the basement membrane. Its major components are type IV collagen and glycoproteins, such as laminin and nidogen. The factors influencing their turnover include not only the synthesis but also degradation. It has been observed that kidney extracellular matrix is composed of, inter alia, several collagens, proteoglycans or glycoproteins [21]. Therefore, in the present study, we evaluated the expression, content and activity of enzymes engaged in ECM constituent degradation in renal tissues [22,23,24,25].

Stromelysines, represented by three enzymes, are a group of MMPs. We compared two of them—MMP-3 (stromelysin-1) and MMP-10 (stromelysin-1)—in human kidney cancer with the parts of the same organ that were unaffected. Such parts were used as control material because taking a kidney from healthy donors was out of the question due to ethical reasons. On the other hand, a post-mortem collection of human kidney results in a significant change in the protein content level [26]. Moreover, it was observed that the compared protein content is significantly lower in comparison to the level measured in a tissue freshly taken from a living organism.

To begin with, the DNA content was evaluated to confirm the active ECM reconstruction. With the use of Burton’s method, a higher content of deoxyribonucleic acid in both grades of kidney tumor in contrast to the control tissue was demonstrated. Furthermore, an increase in the DNA level was observed with an increase in cancer grade. The above outcome may indicate that ECM remodeling intensified during the course of kidney cancer.

Using the ELISA test, total stromelysines contents in kidney samples were measured. The contents of measured stromelisynes were differentiated to a significant extent and depended on the respective cancer grade. Both grades of tumor showed a significantly lower content of both enzymes in comparison with the respective control tissue. We found that the control tissue of human kidney contained a much smaller amount of MMP-3 than MMP-10. This proves that stromelysin-2 has an advantage in the reconstruction of extracellular matrix in healthy kidneys. A significantly lower content of MMP-3 in both cancer grades in contrast to the distinctive growth of MMP-10 was observed. The above outcomes revealed that no synthesis or secretion inhibition of cells with regard to the investigated metalloproteinases occurred. We may also suggest that malignant and normal renal cells vary in stromelisyne secretion outside of the cell. It seems, that the level of stromelysin-1 synthesis may be similar for both cell kinds; however, cancer cells limit MMP-3 secretion to the extracellular space. It is well-known that the protein turnover inside the cell is much faster than on the outside [19]. On the other hand, the stromelysin-2 content is at a higher level compared with MMP-3 in cancerous cells, but it is still significantly lower compared to the control tissue. We can assume that in these circumstances the cancer cells did not limit the MMP-10 secretion to the extracellular space to such a huge extent as MMP-3.

The presented stromelysines actual activity per kilogram of total protein content enabled us to compare the activity of both analyzed enzymes in investigated tissues. Our results show that MMP-10 is about three times more active in healthy human kidney than MMP-3 and four times more active in both grades of kidney tumor in comparison to healthy tissue. The involvement of metalloproteinases in the permanent process of extracellular matrix remodeling shows that MMP-10 significantly contributes to the maintenance of ECM homeostasis in a normal kidney. It was noted that a substantial decrease in the activity of both enzymes was associated with an increase in cancer progression stage. However, only MMP-10 from renal carcinoma in the G2 phase reached an actual activity similar to the control tissue. Such an increase in the actual activity of stromelysin-2 may be further evidence of its considerable role in carcinogenic processes. In the cells of both grades of tumor, the activity of MMP-3 and MMP-10 was found, but we did not identify any correlation between enzyme expression/activity and tumor progression.

Moreover, we calculated the specific activity of stromelisynes per kilogram of enzyme protein and determined what part of the enzyme was active without any inhibitor bound to the active site of the enzyme. As opposed to actual activity results, MMP-3 shows a much higher specific activity than MMP-10 in both control and cancerous tissues. In the cancer tissue, the activity is significantly lower for both enzymes. Numerous differences in the obtained outcomes may indicate that the values of specific activity significantly differ depending on the grade and the phase of carcinogenic process. All in all, it seems that most of MMP-3, as opposed to MMP-10, stays in an active form without an inhibitory effect of TIMPs. Apparently, the enzymatic activity is exerted to a greater extent by stromelysin-2 than stromelysin-1 proteins.

As can be seen from the Western blot analysis, both of these stromelysines occurred primarily in high-molecular-weight complexes in the control kidney and in both of the cancer grades. The extracellular matrix is a space for the interaction of various proteins, in many cases occurring without any impact on the enzyme activity. Those complexes disappear following the reduction in disulphide bridges, meaning that the bonds joining all components together are quite weak and noncovalent. MMP-3 in a free active form occurring in all control and tumor tissues as a band with a molecular weight of approximately 48 kDa, only after disulphide bonds were reduced. According to our results, stromelysin-2 was present in a free active form in all examined samples as a very narrow and clearly visible band with a molecular weight of approximately 50 kDa.

In comparison with the results obtained by other authors, it was demonstrated that an increase in the expression of MMP-3 in tumor cells positively correlated with the increased expression of MMP-1 [15]. MMP-10 was also evaluated in a study of 103 patients who had a radical surgery due to RCC. The presence of MMP-10 was found in the cytoplasm of tumor cells in 45 patients. It was associated with local aggressiveness as well as a degree of malignancy of the tumor, whereas its expression did not correlate with the tumor size. Therefore, MMP-10 is regarded as a potential therapeutic target to inhibit the invasion and the metastases of renal cell carcinoma [16]. 

Despite a higher content and a much higher actual activity of stromelysin-2 at both kidney cancer stages, its specific activity is significantly lower in both cancerous and normal tissues compared to stromelysin-1. As a result, the stromelysin-2 catalytic ability may be much lower compared to stromelysin-1. On the other hand, we have to take its high content and actual activity into consideration. That is why we cannot exclude the possibility that such differences between the findings for both stromelysines may indicate their specific involvement in a respective period of growth and a tumor differentiation. In addition, the results presented above show that cancerous cells of the G2 kidney tumor may boost the activity of both enzymes. Compared to MMP-3, a higher MMP-10 content, and a lower specific activity showed that many more stromelysin-1 molecules occurred in an active form. Based on the above, we assumed that there were differences related to the regulation of expression and activation, at least with reference to the investigated matrix metalloproteinases in human renal carcinoma. The control specimen was taken from the same kidney, so the possibility of a carcinogenic influence on the metabolism of the entire kidney should be considered. Consequently, the initiation of ECM degradation may be regarded as one of the most important stages in the tumor growth process [27,28,29]. 

Knowledge of the participation of stromelysines in ECM degradation may be important in understanding the pathomechanism of renal cancer development. It seems that our study presents very important outcomes for renal cell carcinoma. Differences in content and activity between both stromelysines show in which direction we should lead our next studies. Our study considers tissue material that seem to be unique, but at the same time, there is still much research to conduct on this subject. If we consider both enzymes as a potential biomarkers of early detection or prognostic factors, we should aim to marking them in blood and urine. We also have to consider stromelysines as potential targets for treatment and try to discover or synthesize their inhibitors. On the other hand, the greatest limitation of this research was the relatively small study group. It is very important to analyze the content and activity of both stromelysines in a larger group of RCC patients. This study expands the findings on the relationship between stromelysines and renal cell carcinoma. However, taking into account the above, further studies are needed to confirm the potential application of stromelysin-1 and stromelysin-2 in RCC.

## Data Availability

The data that support the findings will be available on request underthe corresponding author’s e-mail: jacek.kudelski@umb.edu.pl.
